# Clinical usefulness of presepsin and monocyte distribution width (MDW) kinetic for predicting mortality in critically ill patients in intensive care unit

**DOI:** 10.3389/fmed.2024.1393843

**Published:** 2024-05-20

**Authors:** Luisa Agnello, Anna Maria Ciaccio, Fabio Del Ben, Caterina Maria Gambino, Concetta Scazzone, Aurora Giglia, Giuseppe Biundo, Andrea Cortegiani, Bruna Lo Sasso, Marcello Ciaccio

**Affiliations:** ^1^Department of Biomedicine, Neurosciences and Advanced Diagnostics, Institute of Clinical Biochemistry, Clinical Molecular Medicine, and Clinical Laboratory Medicine, University of Palermo, Palermo, Italy; ^2^Internal Medicine and Medical Specialties “G. D’Alessandro”, Department of Health Promotion, Maternal and Infant Care, University of Palermo, Palermo, Italy; ^3^CRO Aviano, National Cancer Institute, IRCCS, Aviano, Italy; ^4^Department of Laboratory Medicine, University Hospital “P. Giaccone”, Palermo, Italy; ^5^Department of Anesthesia, Intensive Care and Emergency, University Hospital “P. Giaccone”, Palermo, Italy

**Keywords:** sepsis, biomarkers, presepsin, MDW, diagnosis, Procalcitonin, CRP, mortality

## Abstract

**Background:**

In this study, we explored the accuracy of two new sepsis biomarkers, monocyte distribution width (MDW) and presepsin (PSP), compared to traditional ones, C-reactive protein (CRP) and Procalcitonin (PCT), to identify sepsis and predict intra-hospital mortality by analyzing their kinetic at different time points during hospitalization stay.

**Methods:**

We enrolled 104 patients admitted to the intensive care unit (ICU) of University Hospital “Paolo Giaccone”, Palermo. Among these, 30 (29%) had a clinical diagnosis of sepsis. MDW, PCT, CRP, and PSP were evaluated at admission (T0), after 24 h (T24), 48 h (T48), 72 h (T72), at day 5 (T5), and at discharge (TD).

**Results:**

Patients with sepsis displayed higher levels of PCT and PSP than patients without sepsis at each timepoint; differently, CRP displayed statistically significant differences only at T0, while MDW only at T0 and T24. Patients with increasing levels of PSP displayed lower median survival time than patients with decreasing levels; differences reached statistical significance only at 48 h (20 vs. 29 days, log rank test, *p* = 0.046). Interestingly, PSP was an independent predictor of ICU mortality at 48 and 72 h after hospital admission. Also, the kinetic of PSP had prognostic value, with increased values at 48 h after admission being associated with reduced survival.

**Conclusion:**

Our findings support the role of PSP and its kinetic as a predictor of ICU mortality.

## 1 Introduction

Sepsis represents a significant challenge in clinical wards, especially in the intensive care unit (ICU). Its early recognition is hampered because it is not a definite disease but a syndrome with different pathogen and host factor-associated symptoms (1). However, the time of intervention is fundamental, and a delayed diagnosis is associated with high mortality and morbidity.

Although blood culture has been considered the gold standard for diagnosing sepsis for decades, it has several limitations, including low diagnostic sensitivity with a high rate of false negatives, preanalytical issues, and possible contamination (2).

Literature evidence suggests that circulating biomarkers significantly reflect the systemic host response to infection. Thus, they support the Clinicians in appropriately managing the patient with sepsis (3).

Over the years, spasmodic research for discovering the ideal sepsis biomarker has endured, concluding that no single molecule with optimal sensitivity and specificity for sepsis lasts. Thus, the dream of finding “the troponin” for sepsis is slowly fading. However, promising biomarkers for sepsis screening are emerging. Among these, monocyte distribution width (MDW) has gained much attention in the last few years (4–13). It is a measure of the monocyte’s anisocytosis, and it is calculated by the last generation hemocytometers of Beckman Coulter. It has a great advantage over the other sepsis biomarkers being part of the complete blood count. Another interesting biomarker is presepsin (PSP). PSP, also named soluble CD14 subtype, is the N-terminal fragment of soluble CD14 released from monocyte/macrophage upon activation through proteolysis and exocytosis (14, 15).

Both MDW and circulating PSP levels reflect the monocytes/macrophages activation, which represents a key mechanism in sepsis pathogenesis, especially during the early stages (16).

Beyond early detection of sepsis, monitoring sepsis patients is another critical issue. In such a contest, the kinetic of sepsis biomarkers could provide precious information.

In this study, we explored the accuracy of two new sepsis biomarkers, MDW and PSP, compared to traditional ones, C-reactive protein (CRP) and Procalcitonin (PCT), to predict ICU mortality by analyzing their kinetic at different time points during ICU stay.

## 2 Materials and methods

### 2.1 Study design

This is an observational, prospective, monocentric cohort study performed at the ICU in collaboration with the Institute of Clinical Biochemistry, Clinical Molecular Medicine, and Clinical Laboratory Medicine, University Hospital “Paolo Giaccone,” Palermo, Italy.

Eligible patients were all consecutive adults (> 18 years) admitted to the ICU for any cause from January 2023 to July 2023. At ICU admission, patients were classified as sepsis, based on Sepsis-3 consensus criteria, and controls, i.e., patients without sepsis (17). According to Sepsis-3 consensus criteria, sepsis was defined as life-threatening organ dysfunction caused by a dysregulated host response to infection. Organ dysfunction was identified as an acute change in total SOFA (sequential organ failure assessment) score ≥ 2 points consequent to the infection (17). The infection was defined according to clinical, imaging, and laboratory test findings.

Exclusion criteria were (i) age < 18 years; (ii) incomplete data collection; (iii) failure to determine the MDW parameter; (iv) underlying conditions potentially associated with deregulation of the immune system, including AIDS, organ or bone marrow transplantation and hematologic diseases; (v) < 5 days hospital stay.

We recorded demographical, clinical, and laboratory data for each patient. MDW, PCT, CRP, and lactate were evaluated at different time points, i.e., at admission (T0), after 24 h (T24), 48 h (T48), 72 h (T72), at day 5 (T5), and at discharge (TD). At each time, an aliquot of plasma was obtained by centrifugation of whole blood collected in K_3_-EDTA tubes and stored at −80° until PSP analysis.

The study protocol was approved by the Ethics Committee of the University Hospital of Palermo (nr 07/2019) and was performed in accordance with the principles set out in the Declaration of Helsinki. Informed consent was not required because we used residual material, and no interventions were performed beyond ordinary good and standard clinical practices (blood cell volumes and indices measurement). Additionally, confidentiality was guaranteed because all patients’ data were anonymized.

### 2.2 Laboratory analysis

For each patient, we measured MDW, PCT, CRP and lactate as part of routine clinical care, and PSP for only research purpose on residual material. The latter was identified and stored at −80°C after all ordered laboratory tests were performed.

The whole-blood sample collected in the K_3_-EDTA tube of each patient was analyzed on the UniCel DxH 900 hematology analyzer (Beckman Coulter, Inc., Brea, California) within 2 h from the collection, as recommended by the manufacturer. The instrument provides cell blood count, white blood cell (WBC) differential, and nucleated red blood cell enumeration. Moreover, it measures specific cell volume parameters and the distribution of cell volumes, including the MDW. The software version of the DxH 900 was 1.1.0. As previously described, MDW is calculated using Volume, Conductivity, and Scatter (VCS) technologies, as previously described (18). VCS parameters can detect morphologic changes in immature and reactive cells, like the microscopic evaluation of a peripheral blood smear. Once the monocyte population is isolated, a 1D histogram of the monocyte volume values is accumulated on an extended volume range (available internally to the algorithm). The EV range allows monocyte populations with cell volumes exceeding the five-part differential measuring range to be fully developed to compute an accurate MDW standard deviation.

Plasma PSP was measured by a commercially available non-competitive chemiluminescent enzyme immunoassay combined with Magtration^®^ technology using a point of care device in the laboratory. The method is optimized on an automated immunoassay analyzer (PATHFAST, Mitsubishi, Tokyo, Japan) and is easily implementable at the bedside. The sample is incubated with alkaline phosphatase labeled anti-presepsin polyclonal and monoclonal antibodies coated magnetic particles; after removing the unbound substances by Magtration^®^ technology, a chemiluminescent substrate is added.

Serum CRP and PCT levels were measured by the latex-enhanced immunoturbidimetric assay, while lactate levels by colorimetric assay on a Cobas c503 analyzer (Roche Diagnostics International Ltd, Rotkreuz, Switzerland), according to the manufacturer.

### 2.3 Statistical analysis

Statistical analysis and visualization were performed by R version 4.3.2 (2023-10-31). Normality distribution was assessed preliminarily by q-q plot and Shapiro–Wilk test. Quantitative variables were expressed by the median and interquartile range (IQR), while qualitative variables were by absolute or relative frequency. The correlation was evaluated by the nonparametric Spearman test. Differences between two independent groups for continuous or qualitative variables were estimated, respectively, by the Mann–Whitney test and Fisher’s Exact test. Differences between paired samples (different time points) were evaluated by a nonparametric Friedman test, followed, when statistically significant, by the Wilcoxon Signed Ranks Test with Bonferroni’s correction for post-hoc comparisons. Predictors for time to mortality within the ICU were evaluated by Cox regression. Mortality was not restricted to sepsis cause, and it was defined as all-cause mortality. Survival was studied by Kaplan–Meier analysis. Survival curves were compared by the log rank test.

## 3 Results

**FIGURE 1:**
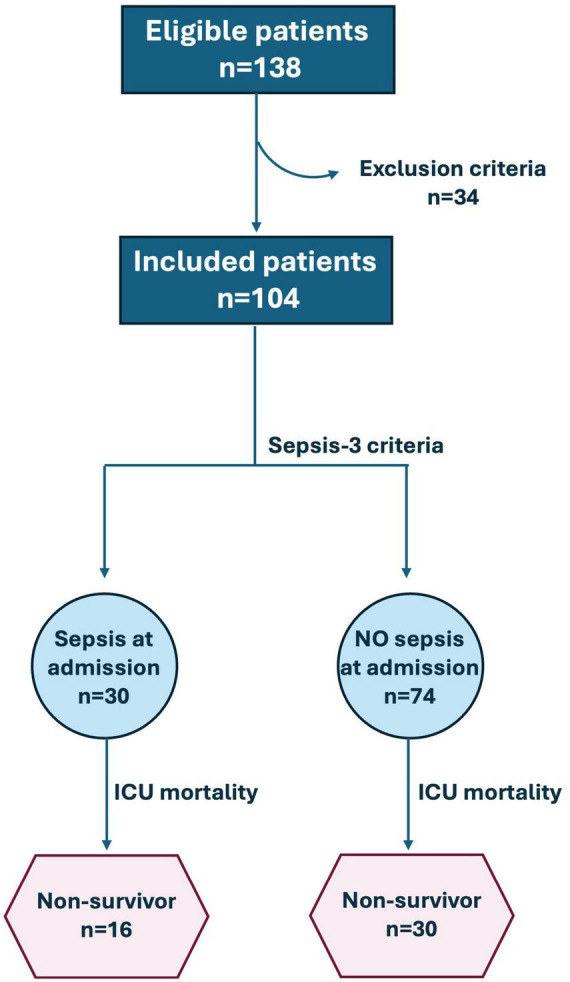
Flow diagram of the study population.

In this study, 104 patients (M:F 56%:44%, median [IQR] age 70 [59–78] years) were enrolled (Figure 1). Among these, 30 (29%) had a clinical diagnosis of sepsis while the remaining (71%) were admitted for the following causes: post-operative (*n* = 20), medical (*n* = 40), surgical (*n* = 3), trauma (*n* = 4), and neurological (*n* = 7). Table 1 reports levels of CRP, PCT, MDW, and PSP measured at 4 timepoints, respectively, basal (t0), at 24 h (t24), 48 h (t48) and 72 h (t72) after the admission at the ICU in the whole study population and subgrouped according to the presence of sepsis. Patients with sepsis displayed higher levels of PCT and PSP than patients without sepsis at each timepoint; differently, CRP displayed statistically significant differences only at t0, while MDW only at t0 and t24 (Table 1). Noteworthy, the half-life of CRP, PCT and PSP are 20–24 h, 12–24 h, and 3–4 h, respectively.

**TABLE 1:** Demographic and biochemical characteristics at different timepoints of the patients investigated.

Variables	All patients	Patients without sepsis	Patients with sepsis	*P*-value
*N* (%)	104	74 (71%)	30 (29%)	
Sex, M:F (%)	56:44%	54:46%	60:40%	0.665
Age, years	70 (59–78)	70 (59–77)	71 (61–80)	0.659
Charlson Comorbidity Index	3.30	3.33	3.52	0.85
Initial SOFA	8 (5–11)	11 (7–12)	7 (4–10)	0.004
**Basal (t0)**				
CRP, mg/L	135.0 (63.1–244.0)	108.5 (53.5–218.0)	185.0 (105.0–296.0)	0.014*
PCT, μg/L	1.55 (0.32–10.40)	1.05 (0.22–4.52)	3.44 (1.05–25.65)	0.003*
MDW	27.6 (25.0–31.7)	25.8 (24.6–30.1)	32.4 (29.1–39.0)	<0.001*
PSP, ng/L	907 (456–1,821)	741 (334–1,573)	1,669 (874–3,209)	0.001*
**After 24 h (t24)**				
CRP, mg/L	170.5 (73.4–277.3)	144.0 (62.2–269.0)	187.5 (138.8–294.5)	0.068
PCT, μg/L	1.73 (0.39–12.10)	1.37 (0.28–6.11)	5.38 (1.40–32.20)	0.002*
MDW	26.6 (23.1–31.9)	25.1 (21.7–29.2)	31.4 (27.4–35.5)	<0.001*
PSP, ng/L	940 (465–2,263)	781 (426–1,755)	1,391 (906–3,567)	0.006*
**After 48 h (t48)**				
CRP, mg/L	159.0 (74.4–239.0)	133.5 (51.8–236.8)	203.0 (93.6–253.0)	0.084
PCT, μg/L	1.90 (0.40–7.15)	0.78 (0.25–3.41)	6.90 (1.22–33.65)	<0.001*
MDW	25.6 (23.2–30.0)	24.7 (23.0–28.9)	27.6 (24.3–31.7)	0.058
PSP, ng/L	1,047 (515–2,799)	767 (420–2,016)	2,060 (868–4,982)	0.004*
**After 72 h (t72)**				
CRP, mg/L	128.0 (52.2–182.0)	117.0 (48.7–177.3)	138.0 (75.2–197.0)	0.236
PCT, μg/L	1.23 (0.36–4.17)	1.07 (0.26–2.56)	3.14 (0.81–13.15)	0.003*
MDW	24.6 (22.2–29.1)	24.6 (22.3–28.8)	26.2 (21.4–29.7)	0.861
PSP, ng/L	1,011 (480–2,835)	774 (334–2,227)	2,150 (754–4,678)	0.005*

CRP, C-reactive protein; MDW, monocyte distribution width; PCT, Procalcitonin; PSP, presepsin.

*Statistical significant.

**FIGURE 2:**
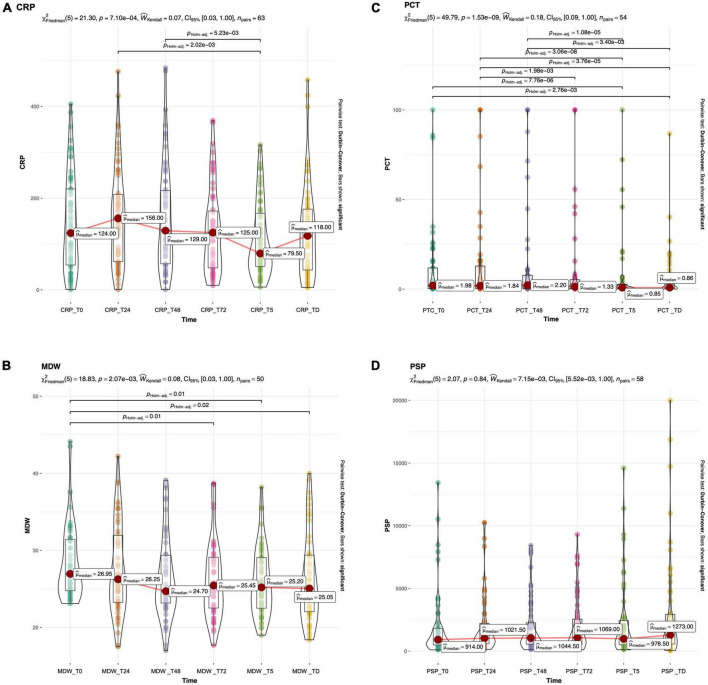
Boxplots with superimposed scatter and density plot of CRP **(A)**, PCT **(B)**, MDW **(C)** and PSP **(D)** at different timepoint in the whole study population. The red dot marks the median value. Bars show significant differences after Holm adjustment for multiple pairwise comparisons.

Data on biomarker trends during the first 72 h, in the whole sample or the sepsis groups, are reported in Table 2. CRP and PCT increased at 24 h, while at 48 h started to decrease (Table 2 and Figure 2); differently, for MDW, a decreasing trend was evident (from t0 to t72), while PSP peaked at 48 h and remained elevated at 72 h (Table 2 and Figure 2).

**TABLE 2:** Timepoint comparisons for the biomarkers investigated.

Variable	All patients	Patients without sepsis	Patients with sepsis
**CRP**	**Overall < 0.001** *	**Overall 0.006** *	**Overall 0.006** *
	t0 vs. t24: 0.048*	t0 vs. t24: 0.042*	t0 vs. t24: > 0.999
	t0 vs. t48: > 0.999	t0 vs. t48: > 0.999	t0 vs. t48: > 0.999
	t0 vs. t72: 0.360	t0 vs. t72: > 0.999	t0 vs. t72: 0.258
	t24 vs. t48: 0.174	t24 vs. t48: 0.414	t24 vs. t48: 0.924
	t24 vs. t72: < 0.001*	t24 vs. t72: 0.012*	t24 vs. t72: 0.042*
	t48 vs. t72: < 0.001*	t48 vs. t72: < 0.001*	t48 vs. t72: < 0.001*
**PCT**	**Overall < 0.001** *	**Overall < 0.001** *	**Overall 0.013** *
	t0 vs. t24: > 0.999	t0 vs. t24: > 0.999	t0 vs. t24: > 0.999
	t0 vs. t48: 0.180	t0 vs. t48: 0.138	t0 vs. t48: > 0.999
	t0 vs. t72: 0.054	t0 vs. t72: 0.066	t0 vs. t72: > 0.999
	t24 vs. t48: < 0.001*	t24 vs. t48: < 0.001*	t24 vs. t48: 0.516
	t24 vs. t72: < 0.001*	t24 vs. t72: < 0.001*	t24 vs. t72: 0.036*
	t48 vs. t72: < 0.001*	t48 vs. t72: < 0.001*	t48 vs. t72: < 0.001*
**MDW**	**Overall < 0.001** *	**Overall 0.012** *	**Overall < 0.001** *
	t0 vs. t24: 0.012*	t0 vs. t24: 0.084	t0 vs. t24: 0.222
	t0 vs. t48: < 0.001*	t0 vs. t48: 0.120	t0 vs. t48: < 0.001*
	t0 vs. t72: < 0.001*	t0 vs. t72: 0.024*	t0 vs. t72: 0.012*
	t24 vs. t48: 0.021	t24 vs. t48: > 0.999	t24 vs. t48: 0.012*
	t24 vs. t72: 0.036	t24 vs. t72: > 0.999	t24 vs. t72: 0.012*
	t48 vs. t72: 0.0192	t48 vs. t72: > 0.999	t48 vs. t72: 0.234
**PSP**	**Overall 0.142**	**Overall 0.243**	**Overall 0.569**

CRP, C-reactive protein; MDW, monocyte distribution width; PCT, Procalcitonin; PSP: presepsin. For each variables the overall *P*-values (Friedman test) and the *p*-values for *post-hoc* comparisons (Wilcoxon Signed Ranks Test with Bonferroni’s correction) are reported. For PSP, post-hoc analysis was not performed due not significant overall test.

*Statistical significant.

Overall mortality within the ICU was 44%, with a median survival time of 26 days (IQR 52–12). Although the sepsis group displayed a higher mortality (52 vs. 40%; Fisher’s *p* = 0.376) and a lower survival time (median 20 vs. 29 days; log rank test *p* = 0.085) than patients without sepsis, the difference was not statistically significant.

**FIGURE 3:**
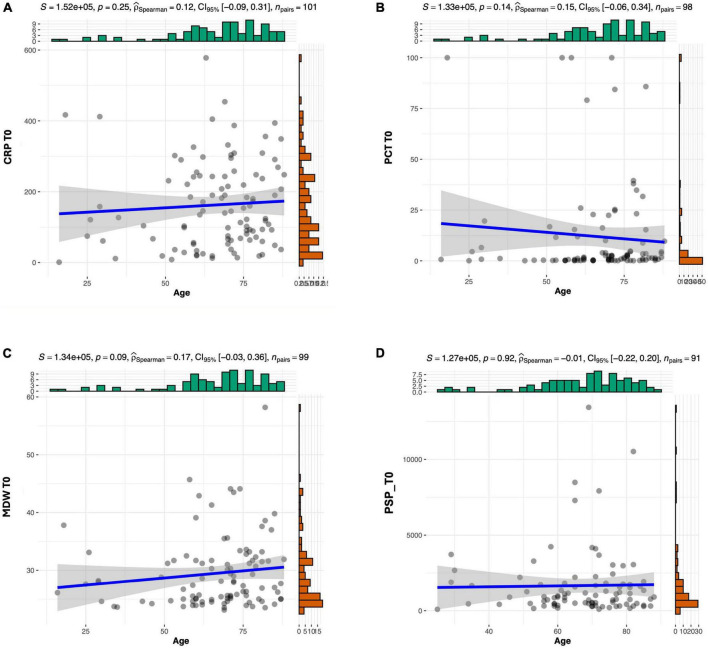
Correlation analysis between age and CRP **(A)**, PCT **(B)**, MDW **(C)**, and PSP **(D)**.

Table 3 reports univariate and multivariate Cox regression for mortality, considering predictors measured at different timepoints. At t0, age was the only independent predictor (*p* = 0.040), while at t24 only MDW (marginally, *p* = 0.05) and at t48 only PSP (*p* = 0.025) (Table 3) were identified. At t72, independent predictors were found to be age (marginally, *p* = 0.050), CRP (*p* = 0.003) and PSP (*p* < 0.001). For PSP and CRP, values have been rescaled to show a meaningful HR, for an increase in 1,000 and 100 units, respectively. Our analysis indicates that age does not exhibit significant correlation with any of the four biomarkers under investigation at admission (Figure 3).

**TABLE 3:** Univariate and multivariate Cox regression for mortality within ICU using predictors at different timepoints (from basal to 72 h).

Predictors at t0	Univariate Cox regression		Multivariate Cox regression	
	**HR (95%CI)**	***P*-value**	**HR (95%CI)**	***p*-value**
Age	1.04 (1.01–1.06)	0.014*	1.03 (1.00–1.06)	0.040*
Sex	1.15 (0.63–2.11)	0.651		
CRP	1.00 (1.00–1.01)	0.057		
PCT	1.01 (1.00–1.03)	0.029*	1.01 (0.98–1.03)	0.660
MDW	1.06 (1.02–1.11)	0.008*	1.06 (0.99–1.14)	0.101
PSP	1.00 (1.00–1.00)	0.122		
SOFA	1.15 (1.05–1.26)	0.002*	1.05 (0.95–1.17)	0.360
**Predictors at t24**				
Age	1.04 (1.01–1.06)	0.014*	1.02 (0.98–1.06)	0.375
CRP	1.00 (1.00–1.01)	0.008*	1.00 (1.00–1.00)	0.890
PCT	1.01 (1.00–1.02)	0.039*	1.00 (0.99–1.02)	0.755
MDW	1.06 (1.02–1.11)	0.009*	1.05 (1.00–1.11)	0.050*
SP	1.00 (1.00–1.00)	0.022*	1.00 (1.00–1.00)	0.273
**Predictors at t48**				
Age	1.04 (1.01–1.06)	0.014*	1.01 (0.98–1.05)	0.470
CRP	1.00 (1.00–1.01)	0.002*	1.00 (1.00–1.01)	0.086
PCT	1.01 (1.00–1.02)	0.045*	1.00 (0.98–1.01)	0.645
MDW	1.05 (0.98–1.11)	0.148		
PSP PSP (1,000 units)	1.00 (1.00–1.00) 1.20 (1.07–1.35)	0.003*	1.00 (1.00–1.00) 1.24 (1.03–1.49)	0.025*
**Predictors at t72**				
Age	1.04 (1.01–1.06)	0.014*	1.04 (1.00–1.07)	0.050*
CRP CRP (100 units)	1.01 (1.00–1.01) 1.75 (1.32–2.32)	<0.001*	1.01 (1.00–1.01) 1.64 (1.20- 2.26)	0.003*
PCT	1.01 (1.00–1.02)	0.169		
MDW	1.06 (0.99–1.13)	0.078		
PSP PSP (1,000 units)	1.00 (1.00–1.00) 1.25 (1.10–1.41)	0.001*	1.00 (1.00–1.00) 1.30 (1.14–1.49)	<0.001*

CRP, C-reactive protein; MDW, monocyte distribution width; PCT, Procalcitonin; PSP, presepsin.

*Statistical significant.

**FIGURE 4:**
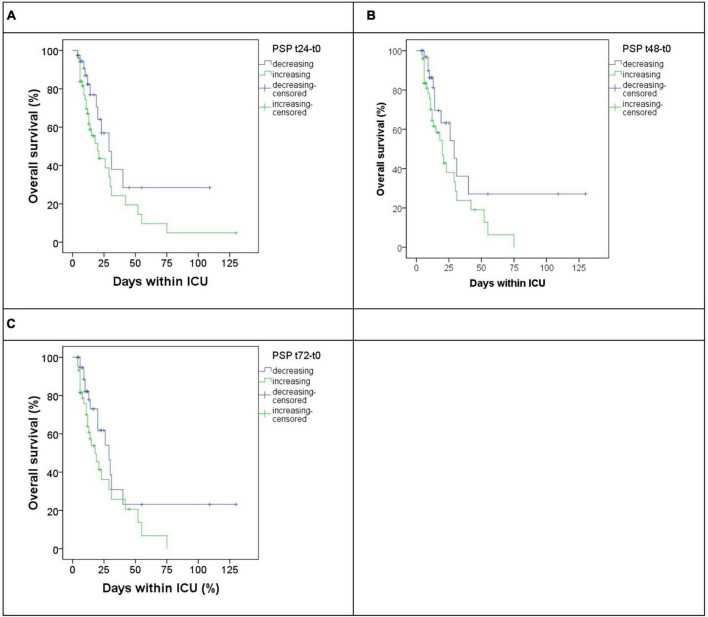
Overall survival in patients subgrouped by increasing (blue) or decreasing (green) gradient of PSP at different timepoints versus basal timepoints, respectively, t24 **(A)**, t48 **(B)**, and t72 **(C)**.

To evaluate a possible role of biomarker kinetic in predicting mortality up to 72 h, 3-time gradients were calculated for each biomarker as [(tX-t0)/t0], where tX indicates the level of the biomarker at 24, 48, and 72 h, while t0 the basal level at admission. Only the gradient [(t48-t0)/t0] for PSP was found to be associated, at the Cox regression, with mortality (Table 4). Based on the results of Cox regression for both PSP point estimates (Table 3) and gradients (Table 4), PSP was further evaluated. PSP gradients at 24, 48, and 72 h were dichotomized into positive (increasing PSP levels) and negative (decreasing PSP levels) values and a Kaplan–Meier analysis was run (Figure 4). Patients with increasing levels of PSP values displayed lower median survival time than patients with decreasing levels; differences reached statistical significance only at 48 h (20 vs. 29 days, log rank test, *p* = 0.046) (Figure 4B), but not, although marginally, at t24 (*p* = 0.074) or at t72 (*p* = 0.058) (Figures 4A, C).

**TABLE 4:** Cox regression for mortality within ICU for patients using timepoint gradients of different predictors.

Predictor	Gradient	HR (95%CI)	*p*-value
MDW	(t24-t0)/t0	4.06 (0.78–21.09)	0.096
	(t48-t0)/t0	1.32 (0.13–13.97)	0.816
	(t72-t0)/t0	2.80 (0.35–22.45)	0.332
CRP	(t24-t0)/t0	1.08 (0.78–1.49)	0.648
	(t48-t0)/t0	1.16 (0.96–1.41)	0.133
	(t72-t0)/t0	0.98 (0.92–1.04)	0.451
PCT	(t24-t0)/t0	1.05 (0.93–1.19)	0.433
	(t48-t0)/t0	1.01 (0.99–1.02)	0.514
	(t72-t0)/t0	1.01 (0.99–1.04)	0.240
PSP	(t24-t0)/t0	1.33 (0.90–1.96)	0.157
	(t48-t0)/t0	1.23 (1.02–1.48)	0.031*
	(t72-t0)/t0	1.11 (0.98–1.26)	0.108

CRP, C-reactive protein; MDW, monocyte distribution width; PCT, Procalcitonin; PSP, presepsin.

*Statistical significant.

We determined the performance metrics for PSP at 48 h, PSP gradient at 48 h, and MDW at 24 h using decisional cutoffs optimized to maximize the positive predictive value. This approach was chosen to ensure high confidence in the true positivity of events as indicated by the markers, thereby supporting more decisive actions in clinical settings. At the same time, we compared these results with the initial SOFA score. Results are shown in Table 5. From these results, it seems that biomarker metrics are similar to the initial SOFA. To further investigate the potential additional contribution of the biomarkers with respect to the initial SOFA, we performed a separate multivariate analysis including the score. However, none of the covariates (age, SOFA, PCT, and MDW) maintained a significant association with ICU mortality, suggesting a potential confounding effect, likely due to the limited statistical power of the analysis. As a workaround, we conducted a bivariate analysis taking into consideration each biomarker in turn and SOFA. This analysis revealed that MDW at baseline maintains its significance (*p* = 0.025), as did the SOFA score (*p* = 0.049), suggesting their independent contributions to ICU mortality. Conversely, PCT did not maintain its significance when analyzed in conjunction with the SOFA score (PCT *p* = 0.132; SOFA *p* = 0.009). Similarly, we evaluated MDW at 24 h and PSP at 48 h. Here, MDW at 24 h continued to show significant association, unlike the SOFA score (MDW *p* = 0.044; SOFA *p* = 0.052). For PSP at 48 h, both maintained significance (PSP *p* = 0.020; SOFA *p* = 0.025). These results suggest that the biomarkers evaluated are indeed independent predictors of mortality, with respect to the initial SOFA score.

**TABLE 5:** Performance for PSP at 48 h, PSP gradient at 48 h, and MDW at 24 h for predicting mortality.

	Cut-off	Sensitivity (%)	Specificity (%)	PPV (%)	NPV (%)
PSP at 48 h	2,650	41	87	74	62
PSP at 72 h	2,000	50	80	70	63
PSP gradient 48 h	0.4	51	76	66	63
MDW at 24 h	36	21	91	62	63
Initial SOFA score	10	48	88	75	68

Based on the specified decisional threshold and subsequent analysis, our investigation has identified the potential existence of a categorical biomarker integrating both static and dynamic measurements of PSP levels. We have devised a composite threshold termed PSP score, which registers as positive if either PSP at 48 h exceeds 2650 or PSP gradient at 48 h surpasses 0.4; otherwise, it is considered negative.

The contingency table illustrates the distribution of patient outcomes categorized by their PSP score status, depicting a clear distinction between survival and mortality outcomes (Table 6). Employing Fisher’s Exact Test for Count Data, we determined a *p*-value of 0.005, indicating a statistically significant association between PSP score status and patient outcomes. The odds ratio estimates of 3.79, with a 95% confidence interval ranging from 1.45 to 10.42, further supports this association.

**TABLE 6:** Contingency table of PSP score deemed positive if PSP at 48 h > 2,650 or PSP gradient at 48 h > 0.4.

	Survivor	Non-survivor
PSP score (−)	30	14
PSP score (+)	15	27

In conclusion, our analysis reveals that patients with a positive PSP score exhibit a 3.79-fold higher likelihood of mortality in the ICU compared to individuals with a negative PSP score. This finding highlights the potential clinical relevance of the PSP score as a prognostic indicator for patient outcomes in critical care settings.

## 4 Discussion

With the awareness that the ideal sepsis biomarker does not exist, the research is focused on identifying reliable biomarkers to assist Clinicians in the appropriate patient management, from early detection to prognostication. Although hundreds of sepsis biomarkers are currently under investigation, PSP and MDW are the most promising in terms of both accuracy and advantages over the traditional (9, 19). A recent meta-analysis by Paraskevas et al. (20), including twenty-nine studies, showed that PSP has a good accuracy for diagnosing sepsis with an area under the curve of 0.875 and a sensitivity of 81%. Previous meta-analyses also displayed good diagnostic value of PSP for sepsis (21–24). Similarly, Motawea et al. (25), showed that MDW has good accuracy for diagnosing sepsis, superior to PCT (25). Also, Huang et al. (10) and Agnello et al. (7) performed a meta-analysis to evaluate the diagnostic accuracy of MDW in adult patients with sepsis concluding that MDW is a reliable biomarker of sepsis (7, 10). Although the accuracy for detecting sepsis is comparable to CRP and PCT in most studies, PSP and MDW hold several advantages over CRP and PCT. Indeed, altered PSP and MDW levels indicate monocyte activation, and their increase is independent of the causative pathogen (26, 27). They can be easily measured. In addition, MDW is part of cell blood count (CBC) and, as such, is always available to Clinicians. On the other hand, PSP may be measured at the bedside by a point-of-care analyser. Thus, MDW and PSP have a rapid turn-around time (< 5 and < 20 min, respectively), aligned with the imperative for swift diagnosis, which is critical in acute settings. Finally, both MDW and PSP have low costs.

In this study, we evaluated the absolute value and the kinetic of MDW, PSP, CRP, and PCT at several timepoints during ICU stay. At admission, MDW, PSP, and PCT were significantly increased in patients with sepsis than without, in line with literature evidence. Interestingly, PSP was an independent predictor of ICU mortality at 48 and 72 h after hospital admission. Also, the kinetic of PSP had prognostic value, with increased values at 48 h after admission being associated with reduced survival.

In survival analysis using Cox regression, the hazard ratio (HR) quantifies the effect of a one-unit increase in a predictor variable on the hazard. Evaluating the results, it is crucial to consider the scale of the predictor, especially when it spans a wide range, such as thousands, like PSP does. When a predictor variable covers such extensive values, interpreting the HR for a single unit increase may not be practically informative or may lead to misleading conclusions. For example, the HR of PSP at 48 h is 1.0002, and it may seem irrelevant, but it means that a PSP rise of 1000 corresponds to an HR of 1.20, which means an increase in risk of 20%, which is not negligible. To enhance the interpretability and relevance of the HR in these cases, we also showed the rescaled predictor variable for a value compatible with its dynamic range. For instance, by scaling PSP that varies in the thousands to reflect changes per thousand units, the resultant HR of 1.24 directly conveys the impact of substantial and realistic increments in the PSP, providing a more meaningful understanding of its influence on the hazard. In our opinion this approach not only aids in clearer interpretation but also helps in comparing and communicating the effects of such predictors in a clinically relevant manner.

Thus, our findings support the role of PSP and its kinetic as a predictor of ICU mortality. Previously, some Authors assessed the prognostic value of PSP in patients with sepsis showing its accuracy in predicting mortality (28, 29). However, only a few evaluated its kinetic during a hospital stay. Similarly to our study, Shimoyama et al. (30) measured PSP longitudinally in ICU patients. The authors showed that PSP values on days 3 and 5 after ICU admission were independent predictors of 28-day mortality. The ΔPSP Day 3-Day 1 of ICU stay showed the best performance for predicting mortality compared to absolute values. Similarly, Hassan et al. (31) explored the clinical value of PSP kinetic in critically ill patients, measuring it at admission and the third day of hospitalization. They showed an increased trend from admission to day 3 in non-survivor patients. Also, Masson et al. (32) displayed an increasing trend in PSP levels in non-survivors within the first week of hospitalization.

Overall, these findings indicate the importance not only of evaluating the absolute PSP value but also of monitoring its changes over the first days of hospital stay to predict mortality. Noteworthy, all studies highlight the better prognostic power of PSP for predicting mortality than the other biomarkers, especially CRP and PCT. In a recent meta-analysis including 60 studies and a total of 15,681 critically ill patients with sepsis, Molano-Franco et al. (33) showed that PSP, but not CRP and PCT, was an independent predictor of mortality.

In our effort to develop a categorical variable that captures both the absolute value and the dynamic changes over time, we introduced a PSP score defined as positive by either a high absolute value or a significant gradient (Table 6). This approach resulted in a straightforward and effective cutoff, exploring a new avenue in this area of research. We hope that our findings could serve as a useful reference for other research groups engaged in similar studies. Biomarkers must not be used as a stand-alone test. They should be considered as one piece of a comprehensive approach to the patient with sepsis, based on the integration among laboratory medicine, i.e., CRP, PCT, PSP, and MDW detection, clinical features, and microbiological findings, keeping in mind that each single dowel has limitations and strengths.

Recent literature debunked the myth that PCT is a reliable biomarker of sepsis (34–36). Although thousands of articles have been published on PCT and sepsis, an agreed decisional cut-off has yet to be established. Additionally, PCT levels may significantly increase in several non-infectious inflammatory conditions (37). It has been shown that the evaluation of PCT kinetics has poor diagnostic and prognostic accuracy (38). The strength of PCT is the high association with bacterial infection, with an elevated negative predictive value to rule it out (39). Also, the accuracy of CRP in detecting sepsis is not optimal, aggravated using different decisional cut-offs ranging from 2 to 10 mg/L (40, 41).

The main limitation of this study is that it was a single-center small sample size. Our analysis revealed a statistically significant difference with the current number of participants. This indicates that the observed effect is strong enough to be detected even with a relatively small sample size. We acknowledge that any study with a limited sample size has limitations regarding the generalizability of the results. However, the significant findings obtained serve as a preliminary basis for future research, justifying larger studies that can further explore these observations in more extensive samples. Thus, larger multicentre cohort studies are required to confirm our findings. Another limitation of our study is the absence of detailed patient histories prior to ICU admission, which precludes us from determining whether the sepsis originated in the community or within a hospital setting. It is plausible that the biomarker dynamics differ between these two types of sepsis. Stratifying patients based on the origin of sepsis could have potentially revealed more nuanced patterns and improved the performance of our biomarker analysis. Another limitation of the study is that some patients in the control cohort developed sepsis post-admission potentially diluting the contrast between the groups in the timepoints after baseline. This could lead to an underestimation of the true differences in biomarker levels between groups. Despite this, the observed biomarker differences were statistically significant, suggesting a real, and potentially underestimated difference even in the face of potential cohort contamination. In contrast, the undetected differences might actually be there but not detected due to a decreased power of the study caused by this dilution effect.

In this study, we first compared the accuracy of two new promising sepsis biomarkers, i.e., MDW and PSP, in relation to the traditional ones, i.e., CRP and PCT. Beyond the traditional and widely used biomarkers of sepsis, MDW and PSP could be implemented in clinical practice for detecting and monitoring sepsis, respectively. MDW, being part of the CBC, could help detect sepsis early, also when it is not suspected, while PSP kinetic could help monitor sepsis and identify patients who would benefit from intensive treatment.

## Data availability statement

The raw data supporting the conclusions of this article will be made available by the authors, without undue reservation.

## Ethics statement

The studies involving humans were approved by the Comitato Etico Palermo 1, University of Palermo. The study was conducted in accordance with the local legislation and institutional requirements. The participants provided their written informed consent to participate in this study.

## Author contributions

LA: Conceptualization, Data curation, Writing – original draft. AMC: Conceptualization, Data curation, Writing – original draft. FDB: Statistical analysis, Writing – original draft. CMG: Data curation, Writing – review & editing. CS: Data curation, Writing – review & editing. AG: Formal analysis, Writing – review & editing. GB: Formal analysis, Writing – review & editing. AC: Data curation, Writing – review & editing. BLS: Data curation, Writing – review & editing. MC: Conceptualization, Supervision, Validation, Writing – review & editing.
